# Supplementary material for: Reconciling Mining with the Conservation of Cave Biodiversity: A Quantitative Baseline to Help Establish Conservation Priorities
Source: PLoS One. 2016 Dec 20;11(12):e0168348. doi: 10.1371/journal.pone.0168348 (PMC5173368; doi:10.1371/journal.pone.0168348)
Supplement: S1 Dataset — (ZIP) [file pone.0168348.s002.zip › Taxa/Serra Norte/SN_2013/N5S/Lista N5S-76.pdf]

CAVIDADE N5S-0076

Classe	Ordem	Fam/Outros	Gên/Outros	Espécie	S-U	U-S	Zon.
Arachnida	Acari	Rhagidiidae		sp.20	X		E
Arachnida	Acari	Veigaiidae		sp.1		X	E
Arachnida	Amblypygi	Phrynidae	*Heterophrynus*	*longicornis*		2	E
Arachnida	Araneae	Anapidae		sp.1	X		E
Arachnida	Araneae	Araneidae		jovem	X		E
Arachnida	Araneae	Linyphiidae	*Meioneta*	sp.1	X		E
Arachnida	Araneae	Ochyroceratidae		jovem	X		E
Arachnida	Araneae	Oonopidae		jovem	X		E
Arachnida	Araneae	Pholcidae	*Mesabolivar*	*aurantiacus*	X	X	E
Arachnida	Araneae	Pisauridae		jovem	1	1	E
Arachnida	Araneae	Salticidae		sp.1	X		E
Arachnida	Araneae	Salticidae		sp.12	X		E
Arachnida	Araneae	Scytodidae	*Scytodes*	sp.1	2	1	E
Arachnida	Araneae	Theridiidae	*Episinus*	sp.1	X		E
Arachnida	Araneae	Theridiidae	*Theridion*	sp.1	X		E
Arachnida	Araneae	Theridiosomatidae	*Plato*	sp.1	X	X	E
Arachnida	Araneae	Trechaleidae	*Rhoicinus*	sp.1	4		E
Arachnida	Opiliones	Sclerosomatidae	*Prionostema*	sp.1	5	1	E
Arachnida	Opiliones	Stygnidae	*Protimesius*	*gracilis*	1		E
Entognatha	Collembola	Cyphoderidae		sp.1	X	X	E
Entognatha	Collembola	Entomobryidae		sp.1	X	X	E
Entognatha	Collembola	Entomobryidae		sp.6	X		E
Entognatha	Collembola	Entomobryidae		sp.7	X		E
Entognatha	Collembola	Paronellidae		sp.1	X	X	E
Entognatha	Collembola	Paronellidae		sp.6	X		E
Entognatha	Diplura	Campodeidae		sp.1		X	E
Insecta	Blattodea			jovem	1		E
Insecta	Coleoptera	Carabidae		sp.4		X	E
Insecta	Coleoptera	Carabidae		sp.10	X		E
Insecta	Coleoptera	Carabidae		sp.14	X		E
Insecta	Coleoptera	Dytiscidae		sp.10		X	E
Insecta	Coleoptera	Hydrophilidae		sp.5	X		E
Insecta	Coleoptera	Leiodidae		sp.1	X		E
Insecta	Coleoptera	Staphylinidae	Pselaphinae	sp.2	X	X	E
Insecta	Coleoptera	Staphylinidae	Pselaphinae	sp.19	X		E
Insecta	Coleoptera	Staphylinidae		sp.17	X		E
Insecta	Coleoptera	Staphylinidae		sp.18	X		E
Insecta	Coleoptera	Staphylinidae		sp.25	X		E
Insecta	Coleoptera	Staphylinidae		sp.29	X		E
Insecta	Diptera	Cecidomyiidae	Cecidomyiinae	sp.	X		E
Insecta	Diptera	Culicidae	*Culex*	sp.		X	E
Insecta	Diptera	Phoridae	Metopininae	sp.	X		E
Insecta	Diptera	Psychodidae	Phlebotominae	sp.	X		E
Insecta	Diptera	Tipulidae	Tipulinae	sp.	X	X	E
Insecta	Heteroptera	Dipsocoroidea		sp.	X		E
Insecta	Heteroptera	Hebridae		sp.		X	E
Insecta	Heteroptera	Reduviidae	Emesinae	sp.5	1		E
Insecta	Heteroptera	Veliidae	*Microvelia*	sp.	X	X	E
Insecta	Heteroptera	Veliidae	*Paravelia*	jovem		X	E

Insecta	Homoptera	Cixiidae		jovem		X	E
Insecta	Hymenoptera	Diapriidae		sp.2	X		E
Insecta	Hymenoptera	Formicidae	*Brachymyrmex*	sp.1	X		E
Insecta	Hymenoptera	Formicidae	*Camponotus*	sp.1	X		E
Insecta	Hymenoptera	Formicidae	*Camponotus*	sp.2	X		E
Insecta	Hymenoptera	Formicidae	*Eurhopalothrix*	sp.1	X		E
Insecta	Hymenoptera	Formicidae	*Paratrechina*	sp.1		X	E
Insecta	Hymenoptera	Formicidae	*Paratrechina*	sp.2	X		E
Insecta	Hymenoptera	Formicidae	*Pheidole*	sp.2	X	X	E
Insecta	Hymenoptera	Formicidae	*Solenopsis*	sp.1	X		E
Insecta	Hymenoptera	Formicidae	*Solenopsis*	sp.2	X		E
Insecta	Isoptera	Termitidae	*Nasutitermes*	sp.	X	X	E
Insecta	Lepidoptera	Galacticidae		sp.	X		E
Insecta	Lepidoptera	Gelechioidea		sp.	X		E
Insecta	Lepidoptera	Noctuoidea		sp.5	1	1	E
Insecta	Lepidoptera	Noctuoidea		sp.9	1		E
Insecta	Orthoptera	Phalangopsidae	*Paraclodes*	jovem	2		E
Insecta	Orthoptera	Phalangopsidae	*Phalangopsis*	jovem		1	E
Insecta	Psocoptera			jovem	X		E
Gastropoda	Pulmonata	Subulinidae		sp.5	X		E
Reptilia	Squamata	Sauria		sp.	1		E
